# Pharmacokinetics and pharmacogenetics of the MEK1/2 inhibitor, selumetinib, in Asian and Western healthy subjects: a pooled analysis

**DOI:** 10.1007/s00228-017-2217-3

**Published:** 2017-03-10

**Authors:** Angela W. Dymond, Cathy Elks, Paul Martin, David J. Carlile, Gabriella Mariani, Susan Lovick, Yifan Huang, Ulrike Lorch, Helen Brown, Karen So

**Affiliations:** 1 AstraZeneca, Alderley Park, Macclesfield, Cheshire, SK10 4TG UK; 2 Covance, Early Clinical Biometrics, Leeds, UK; 3 AstraZeneca, Personalised Healthcare & Biomarkers, Innovative Medicines and Early Development Biotech Unit, Darwin Building, 310 Cambridge Science Park, Milton Road, Cambridge, CB4 0WG UK; 4 Sandoz Pharmaceuticals, Holzkirchen, Germany; 5 Early Clinical Development, Innovative Medicines and Early Development Biotech Unit, Da Vinci Building, Melbourn, Royston, Hertfordshire, SB8 6HB UK; 6 AstraZeneca Global Medicines Development, Da Vinci Building, Melbourn, Royston, Hertfordshire, SB8 6HB UK; 7 Phastar Ltd, London, UK; 8 AstraZeneca, Gaithersburg, MD 20878 USA; 9 Richmond Pharmacology Ltd, St George’s University of London, London, SW17 0RE UK

**Keywords:** Selumetinib, Asian, Western, Pharmacokinetics, Pharmacogenetics

## Abstract

**Purpose:**

Emerging data on selumetinib, a MEK1/2 inhibitor in clinical development, suggest a possible difference in pharmacokinetics (PK) between Japanese and Western patients. This pooled analysis sought to assess the effect of ethnicity on selumetinib exposure in healthy Western and Asian subjects, and to identify any association between genetic variants in the *UGT1A1, CYP2C19* and *ABCG2* genes and observed differences in selumetinib PK.

**Methods:**

A pooled analysis of data from ten Phase I studies, one in Asian subjects (encompassing Japanese, non-Japanese Asian and Indian Asian subjects) and nine in Western subjects, was conducted. Key findings were derived from the collective exposure data across doses of 25, 35, 50 and 75 mg selumetinib; primary variables were dose-normalized AUC and C_max_.

**Results:**

PK data from 308 subjects (10 studies) were available for the pooled analysis; genetic data from 87 subjects (3 studies) were available for the pharmacogenetic analysis. Dose-normalized AUC and C_max_ were 35% (95% CI: 25–47%) and 39% (95% CI: 24–56%) higher in the pooled Asian group, respectively, compared with Western subjects. PK exposure parameters were similar between the Japanese, non-Japanese Asian and Indian groups. There was no evidence that the polymorphisms assessed in the genes *UGT1A1, CYP2C19* and *ABCG2* account for observed PK differences.

**Conclusions:**

Selumetinib exposure was higher in healthy Asian subjects compared with Western subjects, and these data provide valuable insight for clinicians to consider when treating patients of Asian ethnicity with selumetinib.

**Electronic supplementary material:**

The online version of this article (doi:10.1007/s00228-017-2217-3) contains supplementary material, which is available to authorized users.

## Introduction

Ethnicity and genetic variability in metabolizing enzymes and transporter proteins can influence the pharmacokinetics (PK) and clinical response to drugs, resulting in variability in the response of individuals from different ethnic groups to standard doses of drugs [1]. This raises the risk of therapeutic failure or adverse drug reactions. Consequently, an assessment of the PK and PD (pharmacodynamic) in key ethnic groups is an important consideration in the registration of new medicines, and relevant international guidance has been adopted by the Food and Drug Administration and European Medicines Agency in this context [2].

Selumetinib (AZD6244; ARRY-142886) is an oral, potent and selective, allosteric MEK1/2 inhibitor [3] dosed twice-daily with a short half-life [4, 5], currently in development for oncology indications [6–8]. Very limited data are available on the administration of selumetinib in Asian (Japanese, non-Japanese Asian and Indian) patients, although studies that include Asian patients are underway examining selumetinib in combination with the epidermal growth factor receptor inhibitor gefitinib (NCT02025114 [9]), osimertinib (selumetinib up to 50 mg twice-daily [10]) or docetaxel NCT02448290 [11]. Emerging patient PK data indicate that there may be a difference in selumetinib PK between Japanese and Western patients (NCT01605916 [12]), therefore we sought to investigate further the potential impact of ethnicity on selumetinib PK.

The PK of selumetinib have recently been assessed in ten Phase I studies in healthy subjects; these included one study in Asian subjects (Study 86 [NCT01960374]) and nine studies in Western subjects (Studies 66 [NCT01635023], 69 [NCT01974349], 71 [NCT02056392], 78 [NCT02322749], 80 [NCT02238782], 81 [NCT02063204], 82 [NCT02063230], 83 [NCT02093728] and 85 [NCT02046850]). Pooled analysis of selumetinib PK from these studies could further characterize any effects that ethnicity may have on selumetinib PK in Western, Japanese, non-Japanese Asian, and Indian Asian subjects.

Any differences in selumetinib PK between Japanese and Western patients could be influenced by metabolizing enzymes and/or transporters. The cytochrome P450 2C19 (CYP2C19) and UDP glucuronosyltransferase family 1 member A1 (UGT1A1) metabolizing enzymes (encoded by the *CYP2C19* and *UGT1A1* genes), and the Breast Cancer Resistance Protein (BCRP transporter protein; encoded by the *ABCG2* gene) have important roles in drug metabolism and uptake, respectively [13–15]. Since selumetinib is a substrate of these enzymes and transporter protein (unpublished data^1^), its metabolism and uptake may be affected, impacting on drug exposure. Studies have shown that these metabolizing enzymes and transporter proteins are polymorphic with allele frequencies that differ between ethnic populations [16–18]. Polymorphisms in genes for metabolizing enzymes and/or transporters among individuals can influence the efficacy and toxicity of some anti-cancer therapies [19]. Hence, it is important to explore whether genetic variation could account for any differences observed in the PK of selumetinib. The precise quantitative contributions of metabolizing enzymes to the clearance of selumetinib are not known but it is evident that CYP3A4 and CYP2C19 [20] as well as direct conjugation are involved.

These analyses may provide valuable insight into dose selection in these ethnically diverse populations, and could guide future clinical studies in patients.

## Subjects and methods

### Study objectives

This pooled analysis of PK and pharmacogenetic data from Phase I, single-dose studies of selumetinib (10 mg and 25 mg capsules were used to deliver 25, 35, 50 and 75 mg dose levels) was conducted in healthy subjects of Asian or Western ethnicity (Online Resource 1). The primary objective of the analysis was to characterize any effects of ethnicity on the exposure of single-dose selumetinib in healthy subjects and to assess dose proportionality in Western and Asian subjects. A secondary objective was to explore whether genetic variants of the *CYP2C19, UGT1A1* and *ABCG2* genes might contribute to any selumetinib PK differences observed between Asian and Western subjects. All the studies included in the pooled analysis were conducted in accordance with the ethical principles outlined in the Declaration of Helsinki and the International Council on Harmonization Good Clinical Practice. Written informed consent was obtained from all subjects prior to any study-related procedures.

### Database construction

The pooled analysis database was constructed using data collected in 10 studies (Online Resource 1). PK data were taken from Study 86 (Asian study) and Studies 66, 69, 71, 78, 80, 81, 82, 83 and 85 (Western studies), while pharmacogenetic data were obtained from Study 86 (Asian study) and Studies 66 and 83 (Western studies). Informed consent for genetic assessment was as follows: Study 66, 21 of 27 volunteers gave consent (one of these volunteers discontinued treatments); Study 83, all 26 volunteers gave consent; Study 86, 45 of 72 volunteers gave consent.

Study 86 (Asian study) was a single-center, open-label, dose-escalation study conducted in the UK to assess the safety, tolerability and single-dose PK characteristics of selumetinib in healthy Japanese subjects (first-generation, born in Japan; expatriate of Japan for <5 years), non-Japanese Asian subjects (first-generation, born in an Asian country other than Japan, but not India; expatriate of their country of origin for <5 years) or Indian ethnicity (first-generation, born in the Indian subcontinent; expatriate of their country of origin for <5 years).

Selumetinib dosing started at 25 mg, with escalation planned to a dose level with exposure equivalent to (and not exceeding) the maximum dose of 75 mg permitted in Western healthy subjects. This maximum dose and associated PK exposure limits were set by AstraZeneca safety committee. In the Western studies, subjects were classified as being of White or Black ethnicity; one subject from Study 66 was identified as Asian, but was excluded from the pooled analysis due to a lack of further information regarding his ethnicity.

Further inclusion criteria for the database included fasted subjects dosed with the selumetinib capsule formulation used for Phase III. This meant exclusion of data from other oral formulations used in study 78 and intravenous formulation used in study 80. Data were included solely from study arms where there was an absence of other treatments (e.g. rifampicin, fluconazole) and from subjects with normal renal and hepatic function.

### Pooled analysis of selumetinib exposure and dose proportionality

A merged dataset containing subject identifier, non-compartmental PK parameters and demographics was constructed. The non-compartmental PK parameters analyzed were: area under the plasma concentration-time curve from time zero to infinity (AUC); area under the plasma concentration-time curve from time 0–12 h (AUC_0–12_); maximum observed plasma concentration (C_max_); time to C_max_ (t_max_); elimination half-life (t_1/2_); apparent oral clearance (CL/F).

The PK parameters AUC, AUC_0–12_ and C_max_ were normalized (abbreviated as DN) for each subject, by dividing the specific PK parameter by the nominal dose (in mg units) and for dose per kg of body weight (abbreviated as DWN) using the following formula: PK parameter value / (nominal dose in mg units / subjects baseline body weight in kg units).

The majority of subjects in the Western studies were of White ethnicity, thus this formed the reference category for comparison. Statistical comparisons of selumetinib PK were undertaken between the following ethnic groups: Western vs All Asian; White vs Black; White vs All Asian; White vs Japanese; White vs non-Japanese Asian; White vs Indian. The comparisons were performed by fitting multivariate models to the PK parameters of interest (e.g. dose-normalized AUC and C_max_), with a stepwise backward selection of the covariates including ethnicity, age, sex, and weight.

Dose-exposure proportionality for AUC and C_max_ was assessed for non-Japanese Asian subjects using data from Study 86, and for the pooled analysis Western group by applying the Power model to data from the pooled analysis: Log(Y) = α + β•log(Dose) + ε, where Y = AUC or C_max_. Intercept and slope estimates (including point estimate, standard error, and 90% confidence interval [CI]) were provided for each parameter, and dose proportionality plots were generated for AUC and C_max_. All statistical analyses were performed using SAS version 9.2.

### Bioanalytical methods

Details of the bioanalytical methods are described in Online Resource 2.

### Analysis of selumetinib pharmacogenetics

The pharmacogenetics component of the pooled analysis sought to explore whether polymorphisms in the *CYP2C19*, *UGT1A1* and *ABCG2* genes were associated with differences in selumetinib PK parameters following drug administration. Five of the six genetic variants analyzed (CYP2C19*2, CYP2C19*3, CYP2C19*17, UGT1A1*6, *ABCG2 421C > A*) were single nucleotide polymorphisms while one genetic variant was a tandem repeat (UGT1A1*28.) Genotyping of the variants was performed using a Luminex *CYP2C19* assay (for CYP2C19*2, CYP2C19*3 and CYP2C19*17), Taqman assays (for UGT1A1*6 and *ABCG2 421C > A*), and fragment analysis (for UGT1A1*28).

Each genetic variant was tested for any association with dose-normalized AUC, AUC_0–12_ or C_max_, using linear regression under an additive genetic model (i.e. phenotypic response per additional copy of the variant allele, where 0, 1 or 2 copies can be present at each locus). For each PK parameter Bonferroni’s correction was used to control for multiple testing when assessing statistical significance. A *p* value below 0.008 (*p* < 0.05 with Bonferoni correction for 6 tests) was considered statistically significant. Given the variation in frequency of the variant alleles, analyses were performed stratified by ethnic group (Asian, White, Black) across the studies. A combined estimate across populations was obtained using random effects meta-analysis to allow for between-population heterogeneity.

Genotype distributions were tested for deviation from Hardy-Weinberg equilibrium using Chi-squared tests. All pharmacogenetic analyses were performed using either StataSE version 14.1 or R statistical software version 3.2.2.

### Safety and tolerability

Safety and tolerability findings obtained from Study 86 were summarized and compared descriptively to the safety and tolerability findings from the studies in Western subjects. Additional safety and tolerability data for the Western studies are reported in detail elsewhere and include references [20–22].

## Results

### Demographics and characteristics

Details of the 10 studies included in the pooled analysis are summarized in Online Resource 1. Selumetinib was administered as a single 25 mg dose (Study 83), a 50 mg dose (Studies 81 and 82) or a 75 mg dose (Studies 66, 69, 71, 78, 80 and 85). In Study 86 (Asian study), administered selumetinib doses were 25 and 35 mg in Japanese subjects, 25, 35 or 50 mg in non-Japanese Asian subjects, and 25 mg in Indian subjects.

Baseline demographics and characteristics are summarized by selumetinib dose in Online Resource 3. Data from 308 subjects (25 mg, *n* = 53; 35 mg, *n* = 27; 50 mg, *n* = 38; 75 mg, *n* = 190) across the 10 studies were included in the pooled analysis. The mean age of subjects was similar across each dose cohort, ranging from 28.5 years in the 35 mg dose group to 41.0 years in the 50 mg dose group. In all dose groups, the majority of subjects were male accounting for 87–100% of the study populations. White and Black subjects were more prevalent in the 25 mg and 75 mg dose groups compared with Asian subjects. Japanese subjects were the most prevalent in the 35 mg group, and non-Japanese Asian subjects were the most prevalent in the 50 mg group.

A total of 91 subjects across Studies 66 (*n* = 20), 83 (*n* = 26) and 86 (*n* = 45) were available for pharmacogenetic assessment. Given observed differences in allele frequencies in the variants being studied between South and East Asian populations, and the potential for population stratification to confound these analyses, Indian Asian subjects (*n* = 3) from the Asian subsample in Study 86 were excluded. As there was no further information on the Asian subject from Study 66 regarding ethnicity, this subject was also excluded, leaving a total of 87 subjects (42 East Asians, 21 Black or African American and 24 White) for the pharmacogenetic analyses.

### Selumetinib PK, by ethnicity

In studies where increased selumetinib exposure was anticipated, a dose lower than 75 mg was used to ensure that the mean selumetinib exposure would not exceed the exposure limit. Exposure data from all ethnicity groups under investigation (i.e. Whites, Blacks, Japanese, non-Japanese Asian and Indians) were only available at the 25 mg dose of selumetinib, therefore the use of dose-normalized exposure data across the collective range of doses tested (25, 35, 50 and 75 mg) better reflected the exposures experienced in the clinic (therapeutic dose and any dose reductions).

In the pooled analysis including all selumetinib doses, the geometric means (geomeans) for dose-normalized AUC and AUC_(0–12)_ were higher in All Asian subjects (79.8 and 61.7 ng.h/mL/mg, respectively) compared with Western subjects (52.4 and 42.9 ng.h/mL/mg) (Table 1). Of the Asian sub-categories, the geomean dose-normalized AUC and AUC_(0–12)_ values for selumetinib were generally similar among Indian (69.5 and 53.5 ng.h/mL/mg), non-Japanese Asian (78.9 and 61.1 ng.h/mL/mg) and Japanese (84.8 and 65.7 ng.h/mL/mg) subjects, although there was a slight increase if comparing that of the Japanese subjects with the Indian subjects. In the Western sub-categories, values were similar among White (51.9 and 42.7 ng.h/mL/mg) and Black (53.4 and 43.3 ng.h/mL/mg) subjects (Table 1). Geomeans for dose-normalized C_max_ followed a similar pattern to AUC and AUC_(0–12)_ across these ethnic groups (Table 1).

**Table 1:** Summary of selumetinib PK parameters by ethnicity

Parameter^*^	White (*N* = 149)	Black (*N* = 87)	Japanese (*N* = 27)	non-Japanese Asian (*N* = 36)	Indian (*N* = 9)	Western (*N* = 236)	All Asian (*N* = 72)
DN AUC, ng.h/mL/mg	51.9 (23.9)^†^	53.4 (26.4)	84.8 (21.0)	78.9 (25.4)	69.5 (16.7)	52.4 (24.9)^‡^	79.8 (23.6)
DN AUC_(0–12)_, ng.h/mL/mg	42.7 (24.2)	43.3 (26.7)	65.7 (24.1)	61.1 (24.6)	53.5 (19.6)	42.9 (25.1)	61.7 (24.6)
DN C_max_, ng/mL/mg	17.7 (33.2)	16.9 (39.6)	28.7 (31.0)	24.3 (32.3)	28.1 (16.0)	17.4 (35.6)	26.3 (30.9)
t_max_, h	1.0 (1, 4)	1.0 (1, 4)	1.0 (1, 3)	1.6 (1, 4)	1.0 (1, 2)	1.0 (1, 4)	1.0 (1, 4)
t_1/2_, h	9.3 (3.11)^†^	8.7 (2.51)	11.5 (3.96)	9.8 (2.58)	9.5 (3.31)	9.1 (2.91)^‡^	10.4 (3.32)
CL/F, L/h	19.9 (5.40)^†^	19.5 (5.71)	12.0 (2.56)	13.0 (2.94)	14.6 (2.32)	19.7 (5.51)^‡^	12.8 (2.81)

*AUC, AUC_(0–12)_ and C_max_ are presented as geometric mean (%CV); t_max_ is presented as median (min, max); t_1/2_, CL/F and are presented as arithmetic mean (SD); ^†^ *N* = 148; ^‡^ *N* = 235

AUC, area under the plasma concentration time curve to infinity; AUC_(0–12)_, AUC from 0 to 12 h; *CL/F*, apparent oral clearance; *C*_*max*_, maximum observed plasma concentration; *DN*, dose normalized; *t*_*max*_, time to maximum plasma concentration; *t*_*1/2*_, elimination half-life; *SD*, standard deviation

**Fig. 1:**
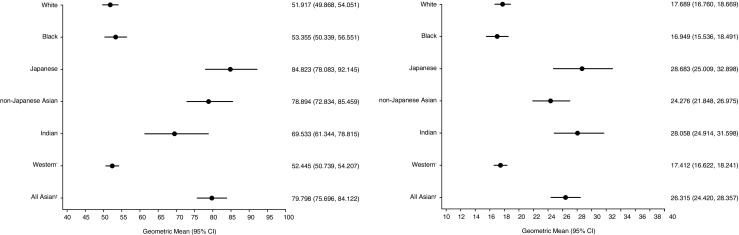
Forest plots of dose-normalized a) AUC and b) C_max_, by ethnicity. ^*^White and Black subjects; ^†^Japanese, non-Japanese Asian and Indian subjects. Data units are: ng.h/mL/mg for AUC; ng/mL/mg for C_max_. White, *N* = 148 for AUC and *N* = 149 for C_max_; Black, *N* = 87; Japanese, *N* = 27; non-Japanese Asian, *N* = 36; Indian, *N* = 9; Western, *N* = 235 for AUC and *N* = 236 for C_max_; All Asian, *N* = 72. AUC, area under the plasma concentration time curve to infinity; C_max_, maximum plasma concentration; DN, dose normalized

In terms of the percentage difference in exposure of all selumetinib doses, geomeans for dose-normalized AUC and C_max_ were higher in the All Asian group compared with the Western group (Fig. 1), by 35% (90% CI: 25–47%) and 39% (90% CI: 24–56%), respectively (Table 2). Of the Asian race sub-categories investigated, Japanese showed the greatest increase in geomeans for dose-normalized AUC compared to White by 51% (95% CI: 35–67%), followed by non-Japanese Asian (37% with 95% CI: 24–52%) then Indian (22% with 95% CI: 3–45%) (Table 2). A different ordering was detected for C_max_: Japanese (45% with 95% CI: 25–69%), followed by Indian (41% with 95% CI: 11–78%) then non-Japanese Asian (20% with 95% CI: 4–38%) compared to White (Table 2). Among the Asian subjects, PK parameters were generally similar between the Japanese, non-Japanese Asian and Indian groups, with differences in the geomeans for dose-normalized AUC and dose-normalized C_max_ ranging from 2 to 18% when comparing non-Japanese Asian and Indian against Japanese (Fig. 1, Table 1). No difference in dose-normalized AUC or C_max_ was observed between White and Black subjects (Table 2).

**Table 2:** Dose-normalized AUC and C_max_, statistical comparisons between ethnic groups

Parameter	DN AUC*			DN C_max_		
	Geometric least squares mean	Difference^†^	95% CI	Geometric least squares mean	Difference^†^	95% CI
All Asian (*n* = 72) vs Western (*n* = 236)	4.284 vs 3.982	1.353	1.246, 1.469	3.205 vs 2.877	1.388	1.235, 1.560
Black (*n* = 87) vs White (*n* = 149)	3.989 vs 3.975	1.014	0.951, 1.081	2.847 vs 2.907	0.942	0.860, 1.032
Japanese (*n* = 27) vs White (*n* = 149)	4.384 vs 3.975	1.505	1.354, 1.673	3.280 vs 2.907	1.453	1.251, 1.687
non-Japanese Asian (*n* = 36) vs White (*n* = 149)	4.292 vs 3.975	1.373	1.244, 1.516	3.088 vs 2.907	1.198	1.042, 1.377
Indian (*n* = 9) vs White (*n* = 149)	4.177 vs 3.975	1.223	1.034, 1.447	3.247 vs 2.907	1.405	1.108, 1.781

*Western, *n* = 235; White, *n* = 148; ^†^Point estimate: adjusted ratio of geometric means; for each comparison, a value above one implies an increase in exposure versus the comparator

Data from the following studies: 66 [NCT01635023], 69 [NCT01974349], 71 [NCT02056392], 78 [NCT02322749], 80 [NCT02238782], 81 [NCT02063204], 82 [NCT02063230], 83 [NCT02093728], 85 [NCT02046850] and 86 [NCT01960374]

Data units are: ng.h/mL/mg for AUC; ng/mL/mg for C_max_

*AUC*, area under the plasma concentration time curve to infinity; *CI*, confidence interval; *C*_*max*_, maximum plasma concentration; *DN*, dose normalized

Higher geomean exposures were detected in the All Asian subjects compared to Western subjects when AUC (5229 vs 4126 ng.h/mL/mg/kg), AUC_(0–12)_ (4045 vs 3372 ng.h/mL/mg/kg) and C_max_ (1724 vs 1369 ng/mL) were normalized by dose per kg of bodyweight (Online Resource 4).

This study showed that the median t_max_ of selumetinib peaked at 1.0 to 1.6 h for all ethnic groups while the arithmetic mean terminal t_½_ value was slightly higher in All Asian subjects (10.4 h) compared with Western subjects (9.1 h) (Table 1).

The ANOVA stepwise model fitting procedure identified weight and ethnicity as the key covariates associated with exposure, with age and sex not showing a clear association with exposure. Overall, the PK variability looked similar at all doses of selumetinib, and in each ethnic group.

### Selumetinib dose-exposure proportionality, by ethnicity

**Fig. 2:**
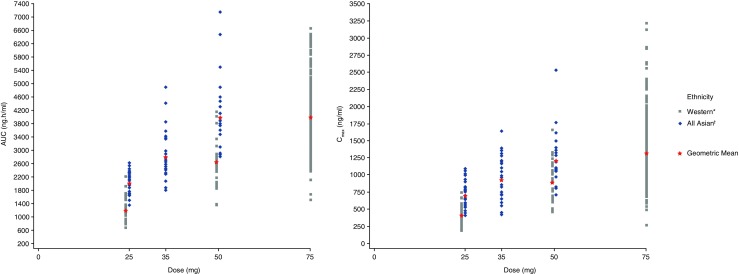
Scatter plot of a) AUC and b) C_max_ against dose, by ethnicity. ^*^White and Black subjects; ^†^Japanese, non-Japanese Asian and Indian subjects. Western and All Asian data points at the 25 and 50 mg doses are staggered to allow greater visual clarity. AUC, area under the plasma concentration time curve to infinity; C_max_, maximum plasma concentration

Scatter plots for individual and geomean AUC and C_max_ versus selumetinib dose enabled visual assessment of dose-exposure proportionality (Fig. 2). Approximate dose proportionality was observed between single doses of 25 to 75 mg selumetinib in the Western group, whilst approximate dose proportionality between 25 to 50 mg selumetinib was observed in the non-Japanese Asian group (Online Resource 5).

Among the Asian subjects, dose proportionality was statistically analyzed only for the non-Japanese Asian group, since this was the only cohort who received three selumetinib dose levels in Study 86. In this non-Japanese Asian group, statistical dose-exposure proportionality was confirmed for AUC over the dose range studied of 25–50 mg (slope estimate 0.99 with 90% CI: 0.760–1.229; AUC values completely within the critical region of 0.678–1.322), although not for C_max_ (slope estimate 1.017 with 90% CI: 0.708–1.326). As C_max_ was slightly greater than dose proportional, log-transformed C_max_ was also analyzed. This confirmed that the increases in C_max_ from 25 to 35 mg, and from 25 to 50 mg, were not directly dose proportional, as the 90% CIs were just outside the critical region (0.80–1.25). In the Western group, statistical dose-exposure proportionality was observed for Ln C_max_ (slope estimate 1.056 with 95% CI: 0.924–1.189) and for Ln AUC (slope estimate 1.099 with 95% CI: 1.005–1.192) where the critical region is 0.797–1.203 (Online Resource 5).

### Selumetinib pharmacogenetics, by ethnicity

**Fig. 3:**
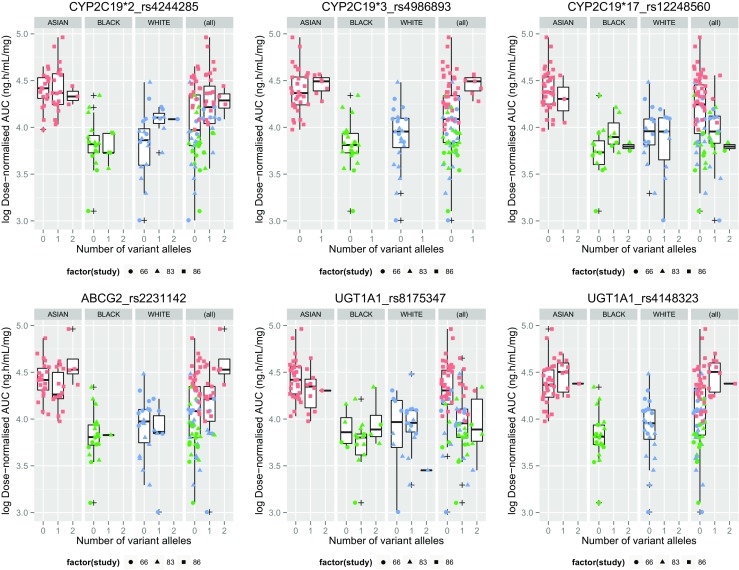
Dose-normalized AUC in genetic variants of *CYP2C19*, *UGT1A1* and *ABCG2*, by ethnicity. AUC, area under the plasma concentration time curve to infinity

**Fig. 4:**
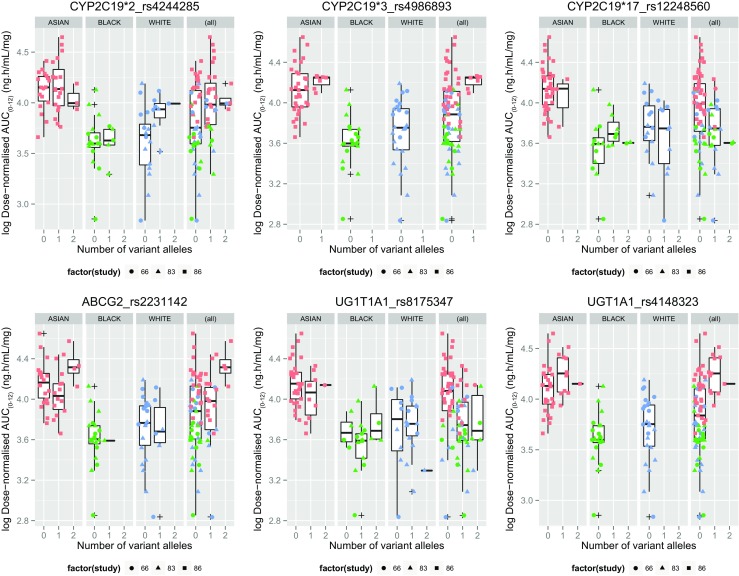
Dose-normalized AUC_(0–12)_ in genetic variants of *CYP2C19*, *UGT1A1* and *ABCG2*, by ethnicity. AUC_(0–12)_, area under the plasma concentration time curve from 0 to 12 h

**Fig. 5:**
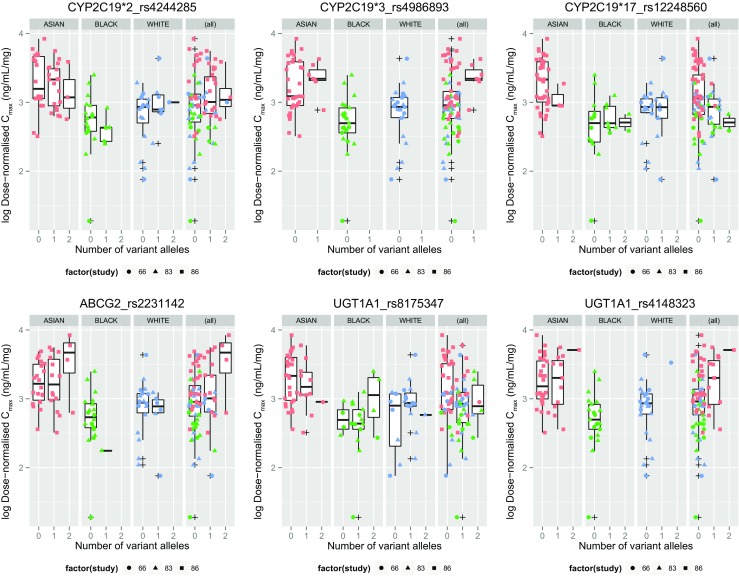
Dose-normalized C_max_ in genetic variants of *CYP2C19*, *UGT1A1* and *ABCG2*, by ethnicity. C_max_, maximum plasma concentration

The distribution of genetic variants for subjects included in the pharmacogenetic analysis, in the Asian, White and Black groups, is summarized in Online Resource 6. Allele frequencies varied between ethnic groups but there was no significant deviation from the genotype frequencies expected under Hardy-Weinberg equilibrium within each population (*p* > 0.05). None of the polymorphisms in the *CYP2C19*, *UGT1A1* and *ABCG2* genes showed any association with dose-normalized values for AUC (Fig. 3), AUC_0–12_ (Fig. 4) or C_max_ (Fig. 5) (Online Resource 7) in any ethnic group after accounting for multiple testing. When subjects of all ethnicities were considered together, the variation of PK parameters between individuals could not be explained by the genetic variants under investigation (Online Resource 8).

### Safety and tolerability

In Study 86 (Asian study), there were no safety or tolerability concerns at any dose level (25, 35 and 50 mg); no subject experienced a serious adverse event (AE) and no AE led to the discontinuation of selumetinib. The number of AEs was highest in the non-Japanese Asian subject group with 50 mg selumetinib while similar numbers of events were reported in each ethnic group at the 25 and 35 mg doses. However, there were no Japanese or Indian subjects who were dosed at 50 mg to make comparisons between the Asian sub-categories. Of the 21 AEs reported, 14 were deemed related to selumetinib by the Investigator. All the AEs were of mild severity except for a single incidence of moderate nausea in a subject who was also experiencing vomiting (mild) at the same time.

These data are consistent with the studies of selumetinib in Western healthy subjects [20–22]. In these studies, there were no serious AEs in Western healthy subjects receiving selumetinib and the majority of AEs were mild and generally considered unrelated to selumetinib. No subjects discontinued selumetinib due to an AE.

## Discussion

This pooled analysis of selumetinib PK and pharmacogenetic findings in Asian and Western healthy subjects explored the impact of ethnicity on selumetinib exposure. The findings provide insight into potential selumetinib dose selection in patients and could be used to guide future studies. The data was analyzed by comparison of exposures in different ethnicities of un-adjusted and dose-normalized PK parameters. This was considered sufficient to provide the required understanding. It is acknowledged that a population PK approach would also have been an appropriate approach. The same bioanalytical method was used for all studies to minimize variability and possible bias in the evaluation. Only data obtained from subjects receiving the same oral formulation as used in Phase III studies were included in the analysis again with intent to minimize variability and possible bias due to formulation or route of administration.

Notably, higher exposures were detected in the Asian subjects compared with Western subjects when AUC and C_max_ were normalized for dose only, and when normalized by dose per kg of bodyweight. This suggests that higher exposures in Asian subjects are not the result of bodyweight differences alone. Moreover, dose-normalized selumetinib exposure was approximately 35–39% higher in Asian subjects (Japanese, non-Asian Japanese or Indian) compared with Western subjects (White or Black) following a single selumetinib dose in the range of 25 to 75 mg. This finding will be taken into consideration when treating patients of Asian ethnicity as the current therapeutic dose is 75 mg twice-daily for all patients. When data are available from patients, safety/exposure relationships for the different ethnicities can be explored. In addition, the apparent differences from the current study will be considered alongside safety, efficacy and PK in patient studies to determine suitable dose levels and regimes. The small departure from direct dose proportionality observed in this study is not considered to be clinically relevant.

Overall, the generally similar PK exposure between the Japanese, non-Japanese Asian and Indian groups suggest that selumetinib exposure is likely to be similar across Asian populations. Similar exposure was observed in the White and Black populations with no statistically significant differences noted in PK findings; however, it should be noted that the studies included in this retrospective pooled analysis were not initially designed to investigate differences in exposure between White and Black populations.

Genetic variants in the *CYP2C19*, *UGT1A1* and *ABCG2* genes did not appear to account for observed PK differences between individuals, suggesting that other genetic or non-genetic factors may be involved, or that there was not enough power to detect such associations.

There were no safety or tolerability concerns in Asian subjects in Study 86; this is similar to the case in the Western studies [20–22]. Furthermore, preliminary data from the TATTON study, which includes Asian patients, indicated no safety concerns in patients who received selumetinib (escalated to a dose of 50 mg twice-daily) in combination with osimertinib [10].

While the data from our analyses provide important insights into the potential impact of ethnicity on selumetinib exposure, a number of limitations of the study were identified. Asian and Western PK data were collected from separate studies hence inter-study and temporal confounding variables are not accounted for. The Western PK data were collected from a wide range of healthy subject studies conducted in different sites in the UK and USA, while the Asian PK data were from a single study conducted in the UK. Variability among the methodologies from the data sources limit the conclusions that could be drawn from the pooled data set. Similarly, dose proportionality could only be assessed in a limited dose range (non-Japanese Asian subjects between 25 and 50 mg; Western subjects between 25 and 75 mg) as exposure limits were set for investigations in healthy subjects for selumetinib, again restricting the conclusions that could be drawn from these data. Plasma concentration data was collected for the active N-desmethyl metabolite and PK parameters derived for the respective individual studies. As the proportion of metabolite relative to parent was low and similar proportions were observed across ethnicities, the metabolite data are not reported in the current manuscript.

Finally, the pharmacogenetic analysis had a small sample size, therefore the findings should be considered as exploratory only since these analyses may be underpowered. The possibility that genetic variation at these loci could have an effect on PK parameters cannot be excluded.

In conclusion, this pooled analysis found that selumetinib exposure from single doses of selumetinib was higher in healthy Asian subjects compared with Western subjects in the dose ranges that are clinically relevant. This is important information for clinicians to take into consideration when treating patients of Asian ethnicity with selumetinib.

## Electronic supplementary material

ESM 1 (DOCX 268 kb)

ESM 2 (JPEG 405 kb)

High Resolution Image (EPS 2792 kb)

ESM 3 (JPEG 164 kb)

High Resolution Image (EPS 1399 kb)

## Footnotes

1 Report nos. Be000021–21 and D6244 KMN036, data on file, AstraZeneca, Cheshire, UK
