# Supplementary material for: Pharmacokinetics and pharmacogenetics of the MEK1/2 inhibitor, selumetinib, in Asian and Western healthy subjects: a pooled analysis
Source: Eur J Clin Pharmacol. 2017 Mar 10;73(6):717–26. doi: 10.1007/s00228-017-2217-3 (PMC5423974; doi:10.1007/s00228-017-2217-3)
Supplement: Supplementary file 1 — (DOCX 268 kb) [file 228_2017_2217_MOESM1_ESM.docx]

# Online Resources

**Online Resource 1** Studies included in the PK pooled analysis

| **Study** | **Study number / NCT number** | **Study arm(s) used** | **N** | **Selumetinib dose, mg** |
| --- | --- | --- | --- | --- |
| **Study 66**  Comparison of Phase II and Phase III capsules; USA | (D1532C00066 / NCT01635023) | Phase III capsules | 26 | 75 |
| **Study 69**  Assessing effects of food; USA | (D1532C00069 / NCT01974349) | Fasted | 34 | 75 |
| **Study 71**  Assess effects on QTc interval; USA | (D1532C00071 / NCT02056392) | All | 50 | 75 |
| **Study 78**  Relative bioavailability of process variants; UK | (D1532C00078 / NCT02322749) | Phase III capsules | 46 | 75 |
| **Study 80**  Assessing absolute bioavailability; UK | (D1532C00080 / NCT02238782) | All | 12 | 75 |
| **Study 81**  Renal impairment vs healthy subjects; USA | (D1532C00081 / NCT02063204) | Healthy subjects | 12 | 50 |
| **Study 82**  Hepatic impairment vs healthy subjects; USA | (D1532C00082 / NCT02063230) | Healthy subjects | 8 | 50 |
| **Study 83**  Effects of CYP3A4 inhibitor itraconazole / CYP2C19 inhibitor fluconazole; USA | (D1532C00083 / NCT02093728) | Selumetinib alone | 26 | 25 |
| **Study 85**  Effects of CYP3A4 inducer rifampicin; USA | (D1532C00085 / NCT02046850) | Selumetinib alone | 22 | 75 |
| **Study 86**  Dose escalation in Japanese, non-Japanese Asian and Indian subjects; UK | (D1532C00086 / NCT01960374) | All | 72 | 25, 35, 50 |

PK, pharmacokinetics

**Online Resource 2** Bioanalytical methods

Bioanalysis was performed by Covance, USA. Selumetinib was analysed in plasma with K_2_EDTA anticoagulant with a range of 2–2000 ng/ml. After addition of deuterated internal standard, samples were extracted by protein precipitation by the addition of 4 volumes of methanol:acetonitrile. After centrifugation, the supernatants were extracted to dryness under nitrogen at 50°C and reconstituted in methanol;water before analysis. Extraction recovery was approximately 93%. Samples were analyzed within the established frozen storage
(-20°C -80°C) stability periods established for selumetinib (24 months). The typical liquid chromatography system was a Prominence SIL-20AC (Shimadzu, Tokyo, Japan). A Phenomenex Luna C18 column was used and maintained at 30°C. A gradient mobile phase system was used with 0.1% formic acid in water (A) or acetonitrile (B) with a flow rate of 0.500 mL/min and the gradient running from 25% B to 65% B over 2 minutes then increasing to 95% B over 0.1 minutes and held for 0.5 minutes before re-equilibration. The mass spectrometer used was an API 5000 or 5500 triple quadruple mass spectrometer (AB Sciex, Foster City, CA, USA). This was run in positive electrospray, multiple reaction monitoring, with an ionspray voltage of 5500 V and TurboIonSpray temperature of 600°C with transitions of 459.0/397.2 monitored.

**Online Resource 3** Baseline demographics and characteristics for subjects included in the PK pooled analysis

| **Characteristic** | **25 mg dose**  **(N=53)** | **35 mg dose**  **(N=27)** | **50 mg dose**  **(N=38)** | **75 mg dose**  **(N=190)** |
| --- | --- | --- | --- | --- |
| Age, years  Mean±SD | 29.9±7.0 | 28.5±7.2 | 41.0±14.9 | 30.0±8.1 |
| Gender, n (%)  Male  Female | 51 (96)  2 (4) | 27 (100)  0 | 33 (87)  5 (13) | 189 (99)  1 (<1%) |
| Ethnicity, n (%)  White  Black  Japanese  non-Japanese Asian  Indian | 13 (25)  13 (25)  9 (17)  9 (17)  9 (17) | 0  0  18 (67)  9 (33)  0 | 13 (34)  7 (18)  0  18 (47)  0 | 123 (65)  67 (35)  0  0  0 |
| Race category, n (%)  Western^*^  All Asian^†b^ | 26 (49)  27 (51) | 0  27 (100) | 20 (53)  18 (47) | 190 (100)  0 |
| Body weight, kg  Mean±SD | 72.6 ± 10.4 | 67.2 ± 11.0 | 72.5 ± 12.5 | 79.1 ± 9.4 |
| Body mass index, kg/m^2^  Mean±SD | 24.0 ± 2.7 | 22.3 ± 3.0 | 24.8 ± 3.9 | 25.4 ± 2.6 |
| Body surface area, m^2^  Mean±SD | 1.9 ± 0.2 | 1.8 ± 0.2 | 1.8 ± 0.2 | 2.0 ± 0.1 |

^*^White and Black subjects
^†^Japanese, non-Japanese Asian and Indian subjects

PK, pharmacokinetics; SD, standard deviation

**Online Resource 4** Forest plots of dose normalized by body weight a) AUC, b) AUC_(0–12)_ and c) C_max_, by ethnicity

*
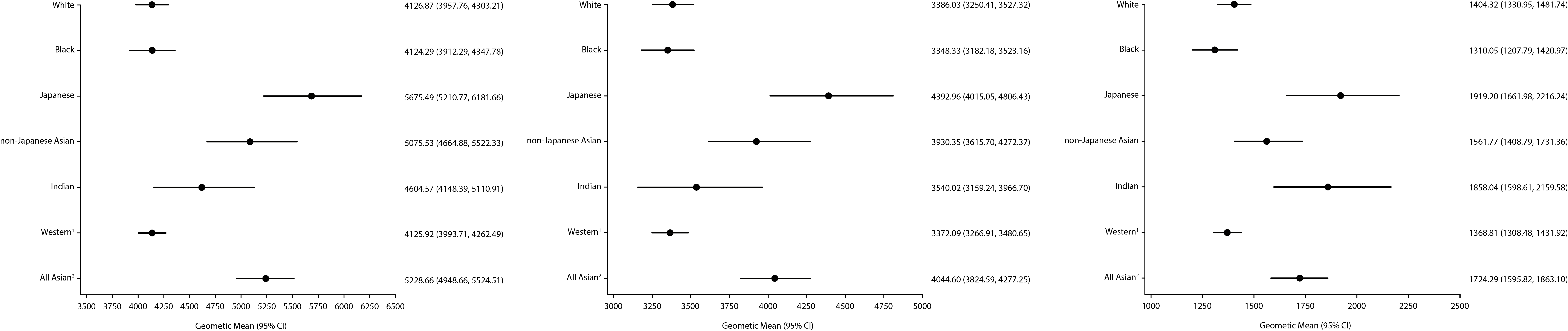

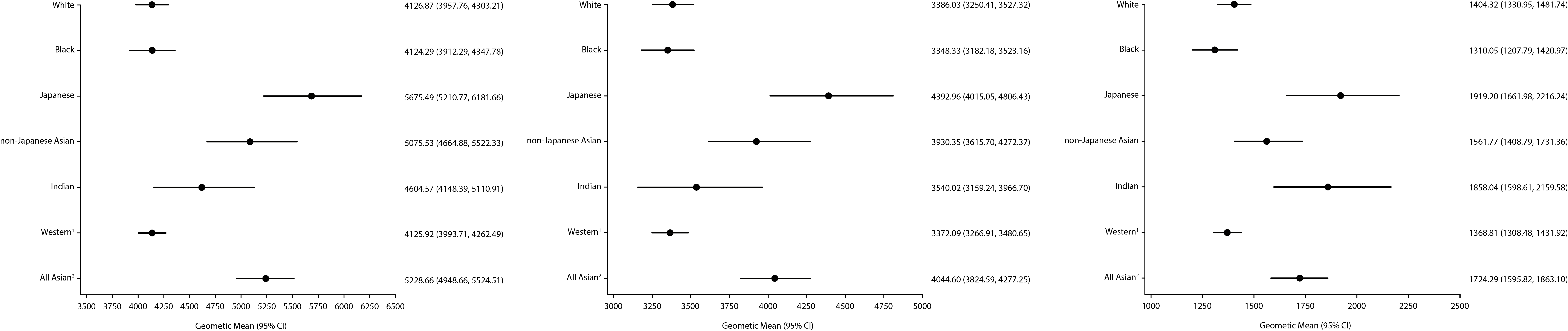
a) DWN AUC b)* **DWN AUC_(0–12)_ c***) DWN C_max_*

^1^White and Black subjects; ^2^Japanese, non-Japanese Asian and Indian subjects

AUC, area under the concentration time curve to infinity; AUC_(0–12)_, AUC from 0–12 hours; C_max_, maximum plasma concentration; DWN, normalized by dose per Kg of bodyweight

Data units are: ng.h/mL/(mg/kg) for AUC and AUC_(0–12)_; ng/mL/(mg/kg) for C_max_

**Online Resource 5** Scatter plot of a) AUC and b) C_max_ against dose, in Western subjects

*
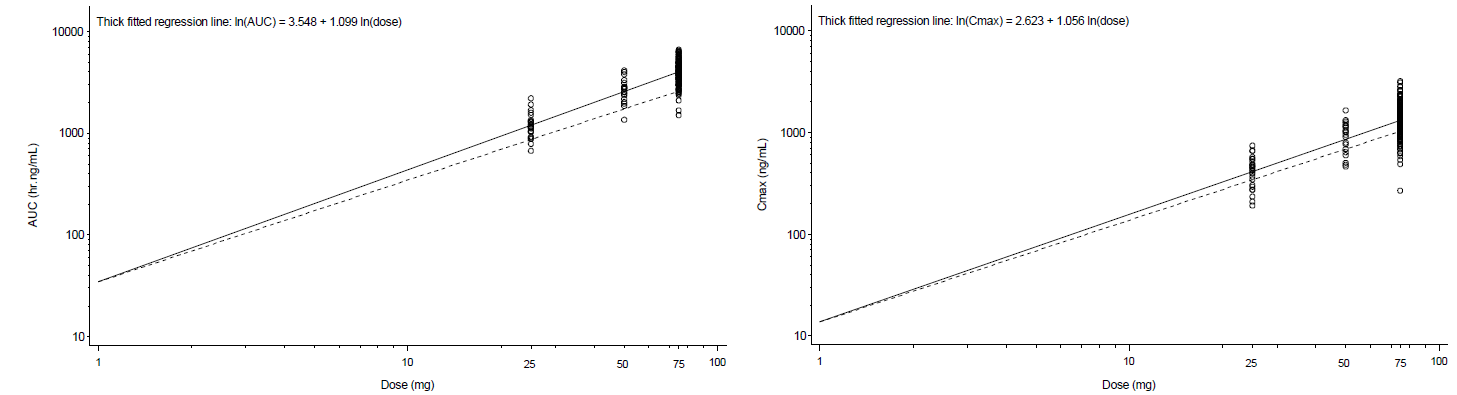

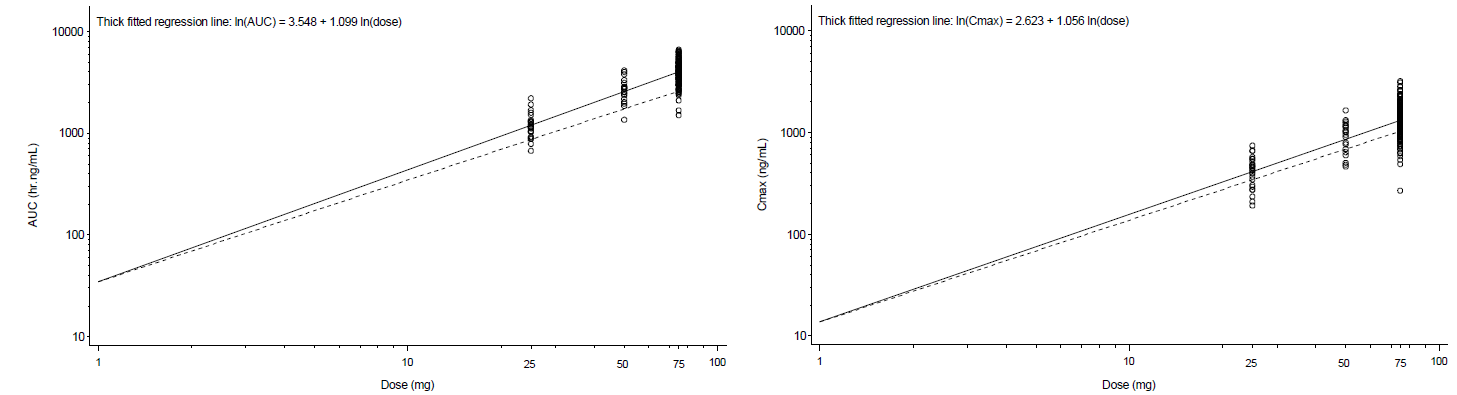
a) AUC b) C_max_*

Solid line is the fitted regression line based on the data; thin dash line represents a line with perfect dose-proportionality, i.e., slope of 1
AUC, area under the concentration time curve to infinity; C_max_, maximum plasma concentration

**Online Resource 6** Distribution of genetic variants for subjects included in the pharmacogenetic analysis

|  | | | **Observed genotype frequencies (number of variant allele copies; MAF)** | | | | | | | | | | | |
| --- | --- | --- | --- | --- | --- | --- | --- | --- | --- | --- | --- | --- | --- | --- |
|  |  |  | **White (N=24)** | | | | **Black (N=21)** | | | | **Asian (N=42)** | | | |
| **Variant** | **rs number** | **Alleles** | **0** | **1** | **2** | **MAF** | **0** | **1** | **2** | **MAF** | **0** | **1** | **2** | **MAF** |
| *CYP2C19*2* | rs4244285 | G/A | 16 | 7 | 1 | 0.188 | 16 | 5 | 0 | 0.119 | 18 | 20 | 4 | 0.333 |
| *CYP2C19*3* | rs4986893 | G/A | 24 | 0 | 0 | 0.000 | 21 | 0 | 0 | 0.000 | 35 | 7 | 0 | 0.083 |
| *CYP2C19*17* | rs12248560 | C/T | 14 | 10 | 0 | 0.208 | 12 | 7 | 2 | 0.262 | 40 | 2 | 0 | 0.024 |
| *ABCG2 421 C>A* | rs2231142 | C/A | 18 | 6 | 0 | 0.125 | 20 | 1 | 0 | 0.024 | 23 | 14 | 5 | 0.286 |
| *UGT1A1*28/37* | rs8175347 | Tandem repeat | 8 | 15 | 1 | NA | 3 | 12 | 6 | NA | 31 | 11 | 0 | NA |
| *UGT1A1*6* | rs4148323 | A/G | 24 | 0 | 0 | 0.000 | 21 | 0 | 0 | 0.000 | 30 | 11 | 1 | 0.155 |

MAF, minor allele frequency; NA, not applicable

**Online Resource 7** Association between genetic variants and pharmacokinetic parameters stratified by ethnicity

|  | **White** | | | | **Black** | | | | **East Asian** | | | |
| --- | --- | --- | --- | --- | --- | --- | --- | --- | --- | --- | --- | --- |
| **Pharmacokinetic parameter** | **N** | **Beta** | **SE** | ***p*** | **N** | **Beta** | **SE** | ***p*** | **N** | **Beta** | **SE** | ***p*** |
| DN AUC (log ng.h/mL/mg) |  |  |  |  |  |  |  |  |  |  |  |  |
| *CYP219C*2* | 24 | 0.198 | 0.115 | 0.101 | 21 | -0.043 | 0.136 | 0.753 | 42 | 0.009 | 0.054 | 0.867 |
| *CYP219C*3* | NA | NA | NA | NA | NA | NA | NA | NA | 42 | 0.063 | 0.092 | 0.497 |
| *CYP219C*17* | 24 | -0.116 | 0.138 | 0.410 | 21 | 0.090 | 0.085 | 0.304 | 42 | -0.104 | 0.162 | 0.525 |
| *ABCG2* | 24 | -0.116 | 0.158 | 0.471 | 21 | 0.018 | 0.272 | 0.947 | 42 | 0.042 | 0.049 | 0.400 |
| *UGTA1*28/37* | 24 | -0.033 | 0.128 | 0.797 | 21 | 0.052 | 0.090 | 0.572 | 42 | -0.153 | 0.075 | 0.048 |
| *UGTA1*6* | NA | NA | NA | NA | NA | NA | NA | NA | 42 | 0.061 | 0.067 | 0.371 |
|  |  |  |  |  |  |  |  |  |  |  |  |  |
| DN AUC_(0–12)_ (log ng.h/mL/mg) |  |  |  |  |  |  |  |  |  |  |  |  |
| *CYP219C*2* | 24 | 0.242 | 0.113 | 0.044 | 21 | -0.010 | 0.136 | 0.943 | 42 | 0.011 | 0.056 | 0.845 |
| *CYP219C*3* | NA | NA | NA | NA | NA | NA | NA | NA | 42 | 0.096 | 0.095 | 0.321 |
| *CYP219C*17* | 24 | -0.127 | 0.140 | 0.372 | 21 | 0.089 | 0.085 | 0.307 | 42 | -0.139 | 0.167 | 0.410 |
| *ABCG2* | 24 | -0.093 | 0.161 | 0.568 | 21 | -0.020 | 0.271 | 0.943 | 42 | 0.043 | 0.051 | 0.405 |
| *UGTA1*28/37* | 24 | -0.039 | 0.130 | 0.769 |  | 0.064 | 0.089 | 0.479 | 42 | -0.177 | 0.077 | 0.027 |
| *UGTA1*6* | NA | NA | NA | NA | NA | NA | NA | NA | 42 | 0.115 | 0.068 | 0.098 |
| DN C_max_ (log ng/mL/mg) |  |  |  |  |  |  |  |  |  |  |  |  |
| *CYP219C*2* | 24 | 0.167 | 0.144 | 0.256 | 21 | -0.110 | 0.225 | 0.630 | 42 | -0.064 | 0.088 | 0.472 |
| *CYP219C*3* | NA | NA | NA | NA | NA | NA | NA | NA | 42 | 0.122 | 0.151 | 0.423 |
| *CYP219C*17* | 24 | 0.041 | 0.169 | 0.812 | 21 | 0.086 | 0.144 | 0.557 | 42 | -0.142 | 0.265 | 0.596 |
| *ABCG2* | 24 | -0.119 | 0.191 | 0.540 | 21 | -0.463 | 0.440 | 0.305 | 42 | 0.104 | 0.080 | 0.200 |
| *UGTA1*28/37* | 24 | 0.190 | 0.150 | 0.217 | 21 | 0.048 | 0.150 | 0.755 | 42 | -0.152 | 0.127 | 0.236 |
| *UGTA1*6* | NA | NA | NA | NA | NA | NA | NA | NA | 42 | 0.073 | 0.110 | 0.511 |

Beta indicates the per-allele change in pharmacokinetic parameter

AUC_,_ area under the concentration time curve to infinity; AUC_(0–12)_, AUC from 0–12 hours; C_max_, maximum observed plasma concentration; DN, dose normalized; NA, not applicable; SE, standard error

**Online Resource 8** Combined pharmacogenetic analysis across ethnicities

| **Pharmacokinetic parameter** | **N** | **Beta** | **95% CI** | ***p*** | **Heterogeneity I^2^** | ***p* for Heterogeneity** |
| --- | --- | --- | --- | --- | --- | --- |
| DN AUC (log ng.h/mL/mg) |  |  |  |  |  |  |
| *CYP219C*2* | 87 | 0.042 | (-0.074, 0.158) | 0.48 | 22.10% | 0.277 |
| *CYP219C*3* | 42 | 0.063 | (-0.117, 0.243) | 0.493 | NA | NA |
| *CYP219C*17* | 87 | 0.004 | (-0.136, 0.144) | 0.956 | 9.70% | 0.33 |
| *ABCG2* | 87 | 0.028 | (-0.063, 0.118) | 0.546 | 0.00% | 0.633 |
| *UGTA1*28/37* | 87 | -0.055 | (-0.188, 0.078) | 0.418 | 36.10% | 0.209 |
| *UGTA1*6* | 42 | 0.061 | (-0.07, 0.192) | 0.363 |  |  |
| DN AUC_(0–12)_ (log ng.h/mL/mg) |  |  |  |  |  |  |
| *CYP219C*2* | 87 | 0.069 | (-0.077, 0.215) | 0.168 | 43.90% | 0.0076 |
| *CYP219C*3* | 42 | 0.096 | (-0.09, 0.282) | 0.312 | NA | NA |
| *CYP219C*17* | 87 | -0.014 | (-0.172, 0.145) | 0.866 | 23.30% | 0.271 |
| *ABCG2* | 87 | 0.029 | (-0.065, 0.123) | 0.544 | 0.00% | 0.711 |
| *UGTA1*28/37* | 87 | -0.058 | (-0.216, 0.101) | 0.476 | 53.00% | 0.119 |
| *UGTA1*6* | 42 | 0.115 | (-0.018, 0.248) | 0.091 | NA | NA |
| DN C_max_ (log ng/mL/mg) |  |  |  |  |  |  |
| *CYP219C*2* | 87 | -0.010 | (-0.155, 0.135) | 0.891 | 4.00% | 0.353 |
| *CYP219C*3* | 42 | 0.122 | (-0.174, 0.418) | 0.419 | NA | NA |
| *CYP219C*17* | 87 | 0.037 | (-0.162, 0.235) | 0.718 | 0.00% | 0.751 |
| *ABCG2* | 87 | 0.015 | (-0.198, 0.228) | 0.887 | 22.80% | 0.274 |
| *UGTA1*28/37* | 87 | 0.016 | (-0.185, 0.216) | 0.878 | 36.10% | 0.209 |
| *UGTA1*6* | 42 | 0.073 | (-0.143, 0.289) | 0.507 | NA | NA |

Results are from a random effects meta-analysis. Beta indicates the per-allele effect on the pharmacokinetic parameter.

AUC, area under the concentration time curve to infinity; AUC_(0–12)_, AUC from 0–12 hours; CI, confidence interval; C_max_, maximum observed plasma concentration; DN, dose normalized
