# Supplementary figures and images for: Pharmacokinetics and pharmacogenetics of the MEK1/2 inhibitor, selumetinib, in Asian and Western healthy subjects: a pooled analysis
Source: Eur J Clin Pharmacol. 2017 Mar 10;73(6):717–26. doi: 10.1007/s00228-017-2217-3 (PMC5423974; doi:10.1007/s00228-017-2217-3)

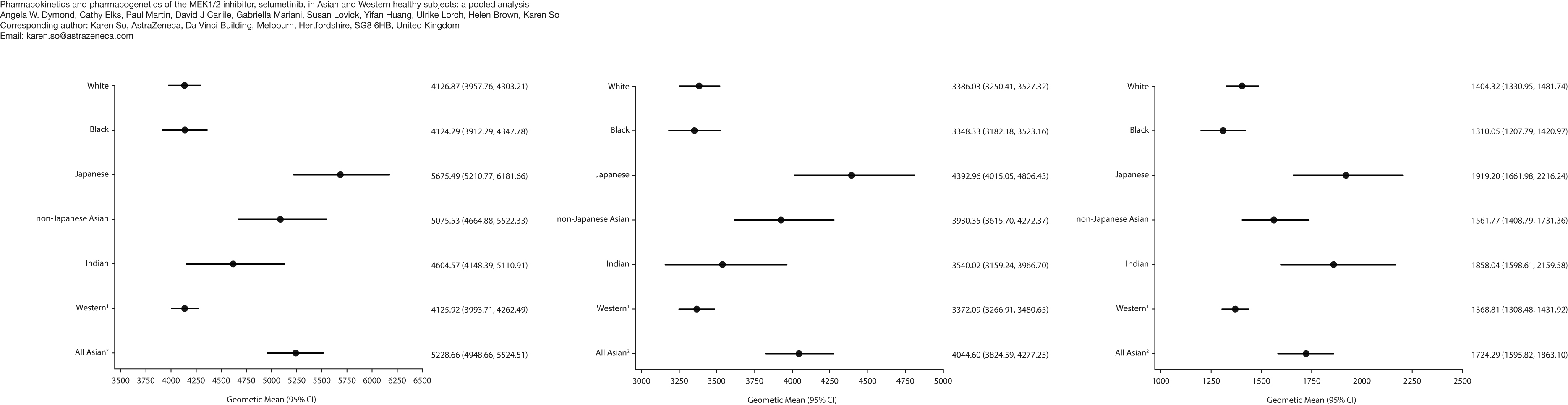

Supplement: Supplementary file 2 — (JPEG 405 kb) [file 228_2017_2217_Fig6_ESM.jpg]

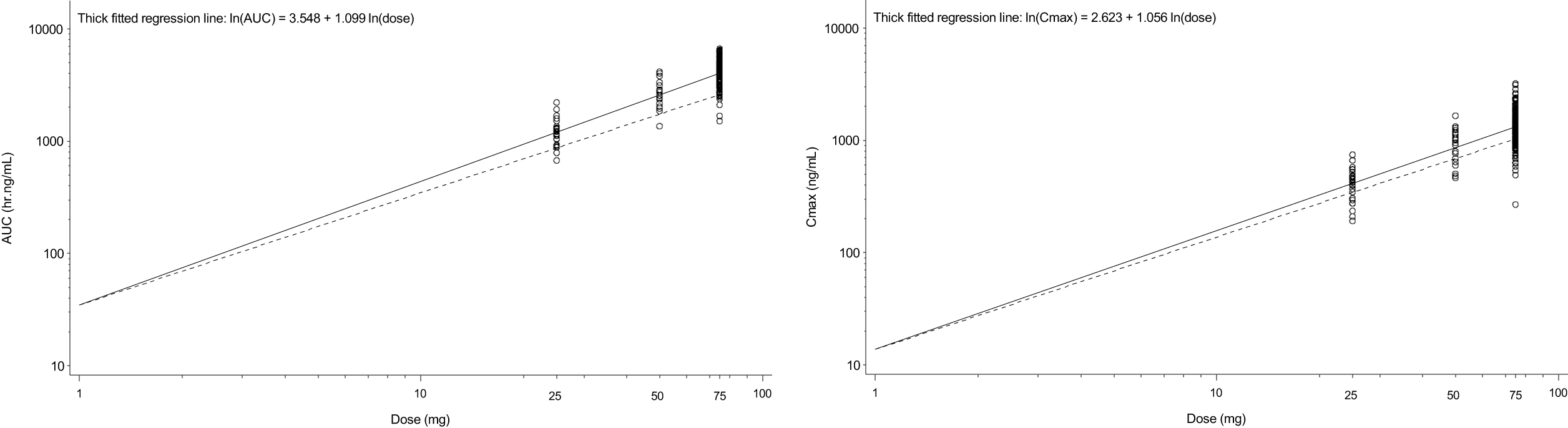

Supplement: Supplementary file 4 — (JPEG 164 kb) [file 228_2017_2217_Fig7_ESM.jpg]
